# Changes in renal medulla gene expression in a pre-clinical model of post cardiopulmonary bypass acute kidney injury

**DOI:** 10.1186/1471-2164-15-916

**Published:** 2014-10-21

**Authors:** Mohamed T Ghorbel, Nishith N Patel, Maimuna Sheikh, Gianni D Angelini, Massimo Caputo, Gavin J Murphy

**Affiliations:** Bristol Heart Institute, School of Clinical Sciences, University of Bristol, Level 7, Bristol Royal Infirmary; Upper Maudlin Street, Bristol, BS2 8HW UK; Deparment of Cardiovascular Sciences, University of Leceister, Leceister, UK; RUSH University Medical Center, Chicago, IL USA

**Keywords:** Acute kidney injury, Cardiopulmonary bypass, Gene expression

## Abstract

**Background:**

Acute kidney injury (AKI) is a common and serious complication of cardiac surgery using cardiopulmonary bypass (CPB). The pathogenesis is poorly understood and the study of AKI in rodent models has not led to improvements in clinical outcomes. We sought to determine the changes in renal medullary gene expression in a novel and clinically relevant porcine model of CPB-induced AKI.

**Results:**

Adult pigs (n = 12 per group) were randomised to undergo sham procedure, or 2.5 hours CPB. AKI was determined using biochemical (Cr51 EDTA clearance, CrCl, urinary IL-18 release) and histological measures. Transcriptomic analyses were performed on renal medulla biopsies obtained 24 hours post intervention or from sham group. Microarray results were validated with real-time polymerase chain reaction and Western Blotting.

Of the transcripts examined, 66 were identified as differentially expressed in CPB versus Sham pig’s kidney samples, with 19 (29%) upregulated and 47 (71%) down-regulated. Out of the upregulated and downregulated transcripts 4 and 16 respectively were expression sequence tags (EST). The regulated genes clustered into three classes; Immune response, Cell adhesion/extracellular matrix and metabolic process. Upregulated genes included Factor V, SLC16A3 and CKMT2 whereas downregulated genes included GST, CPE, MMP7 and SELL.

**Conclusion:**

Post CPB AKI, as defined by clinical criteria, is characterised by molecular changes in renal medulla that are associated with both injury and survival programmes. Our observations highlight the value of large animal models in AKI research and provide insights into the failure of findings in rodent models to translate into clinical progress.

**Electronic supplementary material:**

The online version of this article (doi:10.1186/1471-2164-15-916) contains supplementary material, which is available to authorized users.

## Background

Acute kidney injury (AKI), defined as an acute 25% reduction in glomerular filtration rate [1] is one of the most serious complications post-cardiac surgery with mortality rates of 6–19% [2–4]. The pathogenesis is poorly understood [5] and there have been no advances in the treatment of this condition since the development of dialysis over 30 years ago. Where postoperative renal dysfunction is so severe as to require dialysis, mortality rates are as high as 63% [3]. Cardiopulmonary bypass (CPB) is a major contributor to AKI post-cardiac surgery [6]. Attempts to ameliorate this injury in clinical studies have, to date been unsuccessful [7]. Reno-protective strategies developed in rodent models, the mainstay of research into AKI, have failed to translate into clinical benefits and there is a widely acknowledged need for the development of large animal models of AKI with closer homology to humans [8, 9]. Indeed, it is now accepted that there is a disparity between small rodents and humans explaining why basic research on these models have not yielded great clinical advances [10, 11]. The pig genome-sequencing project is developing fast and providing an important resource to extend the potential of the pig as a biomedical model [12]. We have previously noted qualitative and quantitative similarities between post CPB acute kidney injury in swine [13, 14] and those described in a previous randomised controlled clinical trial of coronary artery bypass grafting with or without CPB [15]. We have also identified medullary hypoxia and an apparently paradoxical elevation in cellular ATP levels in the outer medulla in association with intra-renal vasoconstriction and endothelial dysfunction in kidneys exhibiting AKI as defined clinically at 24 hours post CPB in the swine model [14]. The aim of the current study was to investigate global transcriptomic alterations in the renal medulla in post CPB kidneys in this model in order to gain greater insight into the pathogenesis of this condition.

## Results

### Anaesthesia, monitoring and cardiopulmonary bypass

**Figure 1:**
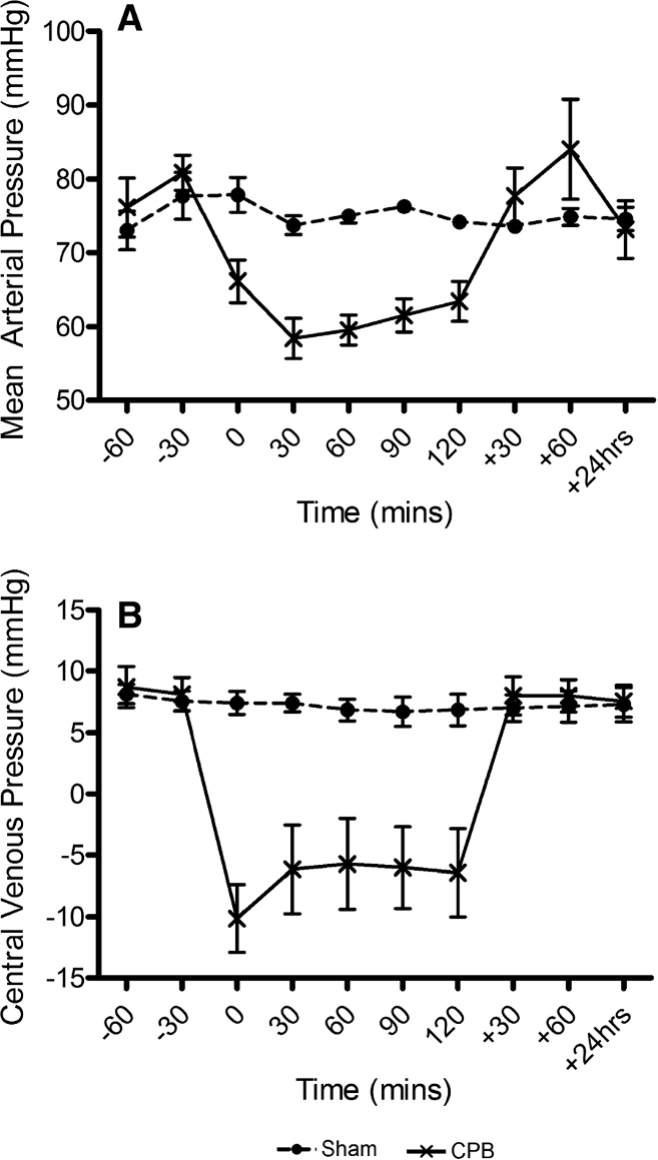
**Haemodynamic changes throughout the experiment. (A)** Mean arterial blood pressure and **(B)** central venous press in the sham group (solid circles) and cardiopulmonary bypass group (cross) over the course of the experiment. Data represents mean (SEM).

All animals survived the study to recovery, re-anaesthesia, re-evaluation and sacrifice. Baseline weight, serum creatinine, urine output and total volume of crystalloid administered over the course of the study were not different between the groups (Table 1). The sham group had a significantly lower level of fractional sodium excretion at baseline suggesting that baseline hydration may have been less in these animals. The mean fractional sodium excretion in both groups was less than 1 in both groups however suggesting that this is unlikely to have confounded our observations. CPB was characterised by a reduction in mean arterial blood pressure (MABP) and circulating haematocrit, as is typically observed clinically. Perfusion pressures were otherwise similar at baseline, immediately post intervention and at 24 hours for both groups (Figure 1).

**Table 1:** **Baseline characteristics**

Variable	Sham (n = 6)	CPB Alone (n = 6)	P value
**Weight (kg)**	58.2 (2.4)	56.9 (1.7)	0.678
**Serum creatinine (mmol/L)**	138.7 (13.2)	134.3 (9.4)	0.794
**Creatinine clearance (ml/min)**	108.6 (8.51)	120.8 (11.25)	0.410
**Urine output (ml/kg/hr)**	1.27 (0.17)	1.65 (0.44)	0.437
**Fractional excretion of sodium (%)**	0.27 (0.08)	0.63 (0.17)	0.080
**Free water clearance (ml/min)**	−0.34 (0.20)	−0.51 (0.17)	0.540
**Protein/Creatinine ratio (mg/mmol)**	21.26 (1.43)	22.41 (2.68)	0.713
**Albumin/Creatinine ratio (mg/mmol)**	0.22 (0.09)	0.08 (0.05)	0.215
**Fluid requirement (ml)**	4833 (105.4)	5500 (223.6)	0.022
**Metaraminol dose (mg)**	0.02 (0.02)	1.7 (0.65)	0.109

Data represents mean (SEM).

### Acute kidney injury

We measured the effect of CPB on renal function as determined by measured Cr51 EDTA clearance and calculated creatinine clearance at 1.5 and 24 hours (Additional file 1: Figure S1). CPB reduced glomerular filtration rate (GFR) by 33.6 ml/hr (95%CI 27.1 to 40.1), p < 0.001 as determined by Cr51 EDTA clearance, and by 54.1 ml/hr (95%CI 22.2 to 86.0), p < 0.001, as determined by creatinine clearance. This effect, equates to stage R in the RIFLE classification of acute kidney injury [1]. The regression coefficient for CrCl vs 51Cr-EDTA clearance was 0.74 at 90 minutes and 0.78 at 24 hours, both p < 0.001. CPB was also associated with significant proteinuria at 24 hours (mean difference: +1.54 (95%CI 1.16 to 2.05) mg/mmol, p = 0.003) and an increase urine IL-18 levels (mean difference: +210.78 (95%CI 127.62 to 293.94) pg/ml, p < 0.001) a specific marker of AKI used in clinical studies. Acute Tubular Necrosis (ATN) was quantified using an established scoring system [16] by a blinded renal histopathologist. This demonstrated no ATN in post CPB kidneys despite the biochemical evidence of acute kidney injury. There was however widespread phenotypic change evident with renal tubular epithelial cells appearing flattened and more epitheloid giving an appearance of tubular dilatation (Mean difference in tubular diameter +2.13 (0.07 to 4.13) μm, p = 0.044).

### Analysis of differentially expressed genes

**Figure 2:**
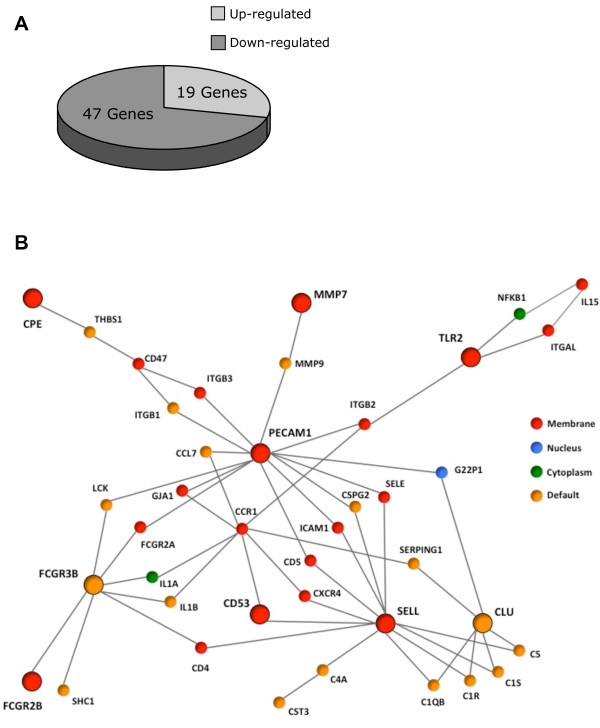
**Differentially expressed genes and gene networks analysis. (A)** Pie-chart of the regulated genes in CPB compared to Sham animals (≥1.6 fold). Three quarters of genes were down-regulated and a quarter up-regulated. **(B)** Biological Association Networks of down-regulated genes. Human literature-confirmed protein-protein interaction map of the identified genes is shown. The big disc nodes are the altered genes. The small nodes are the interacting partners that are not altered in the experiment.

We used the Affymetrix GeneChip Porcine Genome Array in this study as it provides coverage of 20,201 porcine genes (http://www.affymetrix.com/estore). Of the transcripts examined, 66 were identified as differentially expressed in CPB versus Sham pig’s kidney biopsies (*P* < 0.05; 1.6 fold), with 19 (29%) up-regulated and 47 (71%) down-regulated (Figure 2, Table 2). The regulated genes clustered into three classes cell adhesion/extracellular matrix, immune response and metabolic process (Table 3). Most of the upregulated genes belonged to the metabolic process cluster. The downregulated genes belonged to cell adhesion/extracellular matrix and immune response clusters. Collectively, these changes indicate transcriptomic remodelling in the kidney’s medulla following CPB.

**Table 2:** **Genes exhibiting 1.6-fold or greater change of expression in CPB versus Sham animals**

Probe set ID	Symbol	Gene	Fold	P value	Gene ID
Ssc.17934.1.S1_at	STARD10	StAR-related lipid transfer (START) domain containing 10	2.540 up	0.00475	100511029
Ssc.11208.1.S1_at	LOC100625243	Ig kappa chain V-II region RPMI 6410-like	2.413 up	0.0297	100625243
Ssc.7981.1.A1_at	ACOX3	Acyl-CoA oxidase 3, pristanoyl	2.263 up	0.00402	100523005
Ssc.22067.1.A1_at	SLC16A3	Solute carrier family 16, member 3 (monocarboxylic acid transporter 4)	2.223 up	0.00619	100158243
Ssc.15822.1.S1_at	F5	Coagulation factor V	2.079 up	0.00974	397217
Ssc.19237.1.S1_at			2.078 up	0.0433	
Ssc.14462.1.S1_a_at	CKMT2	Creatine kinase, mitochondrial 2 (sarcomeric)	1.988 up	0.0281	733602
Ssc.5607.1.S1_at	ALDH4A1	Aldehyde dehydrogenase 4 family, member A1	1.920 up	0.0458	100820830
Ssc.25191.1.S1_at	LOC100525039	Protein FAM195A-like	1.914 up	0.0414	100525039
Ssc.15778.1.S1_s_at	LOC100736878	Ig kappa chain V-II region RPMI 6410-like	1.827 up	0.00896	100736878
Ssc.4742.1.S1_at	TUBA4A	Tubulin, alpha 4a	1.765 up	0.0323	100151951
Ssc.24386.1.S1_a_at	LOC100620791	Thyroid receptor-interacting protein 6-like	1.737 up	0.0432	100620791
Ssc.8946.1.A1_at			1.714 up	0.0335	
Ssc.5204.1.S1_at	CDA	Cytidine deaminase	1.713 up	0.0169	100515954
Ssc.8646.1.A1_at			1.668 up	0.0079	
Ssc.7654.1.A1_at	LOC100621324	Heat shock-related 70 kDa protein 2-like	1.644 up	0.00441	100621324
Ssc.21893.1.S1_at	LOC100513653	Transmembrane protein C2orf18-like	1.643 up	0.0164	100513653
Ssc.9500.1.A1_at			1.633 up	0.0257	
Ssc.4767.1.S1_at	ACO1	Aconitase 1, soluble	1.606 up	0.00884	100628006
Ssc.14425.1.A1_at			1.606 down	0.0316	
Ssc.13320.1.A1_at	MIR186	MicroRNA mir-186	1.613 down	0.0415	100316566
Ssc.31011.1.A1_at			1.614 down	0.0417	
Ssc.11302.1.S1_at	COL3A1	Collagen, type III, alpha 1	1.617 down	0.0432	
Ssc.8321.1.A1_at			1.627 down	0.0473	
Ssc.18553.1.S1_at			1.627 down	0.0415	
Ssc.5202.1.A1_at	LOC100525593	Uncharacterized LOC100525593	1.628 down	0.0376	100525593
Ssc.11992.1.A1_at	CLU	Clusterin	1.637 down	0.0375	397025
Ssc.25145.1.S1_at			1.669 down	0.0248	
Ssc.14558.1.S1_at	PECAM1	Platelet/endothelial cell adhesion molecule	1.670 down	0.0146	396941
Ssc.6154.1.S1_a_at	LOC100622481	Uncharacterized LOC100622481	1.671 down	0.035	100622481
Ssc.1950.1.A1_at	LOC100513240	GRAM domain-containing protein 1C-like	1.672 down	0.0441	100513240
Ssc.6050.1.A1_at	PECAM1	Platelet/endothelial cell adhesion molecule	1.676 down	0.0439	396941
Ssc.21972.1.A1_at	LOC100523311	T-complex protein 11-like protein 2-like	1.676 down	0.0301	100523311
Ssc.2011.1.A1_at	LOC100152637	Protein kinase C theta	1.677 down	0.0282	100152637
Ssc.15296.2.S1_at	CD53	Leukocyte surface antigen CD53	1.688 down	0.017	100152398
Ssc.3975.2.A1_at			1.694 down	0.0107	
Ssc.12229.1.S1_at	LOC100520933	Cyclin-dependent kinases regulatory subunit 2-like	1.699 down	0.00553	100520933
Ssc.31095.1.A1_at	LOC100624138	Cholinesterase-like	1.700 down	0.0246	100624138
Ssc.30685.1.A1_at	LOC100511051	Protein FAM114A2-like	1.707 down	0.0268	100511051
Ssc.22075.2.A1_at	SELL	Selectin L	1.711 down	0.00383	100127147
Ssc.31071.1.A1_at			1.719 down	0.0136	
Ssc.15283.1.A1_at	KDELR3	KDEL (Lys-Asp-Glu-Leu) endoplasmic reticulum protein retention receptor 3	1.730 down	0.00283	100514523
Ssc.11006.2.A1_at			1.748 down	0.0275	
Ssc.19640.1.A1_at	LOC100152827	Similar to high affinity immunoglobulin E receptor alpha subunit	1.751 down	0.00646	100152827
Ssc.30893.1.A1_at	LOC100511723	PTB domain-containing engulfment adapter protein 1-like	1.807 down	0.0308	100511723
Ssc.28613.1.S1_at			1.839 down	0.0227	
Ssc.11775.1.S1_at	RPS15	Ribosomal protein S15	1.848 down	0.00553	397607
Ssc.21138.1.S1_at	HUS1	HUS1 checkpoint homolog (S. pombe)	1.881 down	0.0022	100192318
Ssc.15540.1.A1_at			1.886 down	0.0338	
Ssc.167.2.S1_a_at	FCGR3B	Fc fragment of IgG, low affinity IIIb, receptor (CD16b)	1.901 down	0.0393	397684
Ssc.4368.3.S1_at	FBXO32	F-box protein 32	1.923 down	0.00578	733657
Ssc.3070.1.S1_at			1.923 down	0.0163	
Ssc.11437.1.A1_at	LOC100525762	DNA damage-inducible transcript 4-like protein-like	1.946 down	0.0388	100525762
Ssc.7122.1.A1_at			1.959 down	0.029	
Ssc.17337.1.S1_at	TLR2	Toll-like receptor 2	2.002 down	0.0452	396623
Ssc.7628.1.A1_at	CSDE1	cold shock domain containing E1, RNA-binding	2.015 down	0.0383	100153226
Ssc.12776.1.A1_at	SELL	Selectin L	2.030 down	0.00984	100127147
Ssc.9217.1.S1_at			2.058 down	0.0425	
Ssc.29716.1.A1_at			2.095 down	0.0449	
Ssc.548.1.S1_a_at	MMP7	Matrix metallopeptidase 7 (matrilysin, uterine)	2.239 down	0.0106	397411
Ssc.24173.1.S1_at	LOC100739731	protein shisa-2 homolog	2.401 down	0.0484	100739731
Ssc.4953.1.A1_at			2.420 down	0.00875	
Ssc.8868.1.S1_at	FCGR2B	Fc fragment of IgG, low affinity IIb, receptor (CD32)	2.527 down	0.0178	613131
Ssc.5464.1.A1_at	CPE	Carboxypeptidase E	2.657 down	0.0126	100037304
Ssc.31013.1.A1_s_at			2.666 down	0.00663	
Ssc.9778.1.S1_at	LOC100049692	Proteoglycan 1 precursor-like	3.328 down	0.0377	100049692
Ssc.10606.1.S1_at			4.615 down	0.00588	
Ssc.16377.1.A1_at	GSTA2	Glutathione S-transferase alpha 2	8.984 down	0.00835	396850

**Table 3:** **Functional pathways represented by the genes altered in CPB versus Sham animals**

Function	Molecules
Immune response	CLU, CD53, FCGR3B, TLR2, FCGR2B, PECAM1
Cell adhesion/extracellular matrix	FACTOR V, TUBA4A, COL3A1, CLU, PECAM1, SELL, MMP7
Metabolic process	STARD10, ACOX3, SLC16A3, CKMT2, ALDH4A1, CDA, ACO1, CHOLINESTERASE-LIKE, CPE, GSTA2, KDELR3

### Gene networks analysis

The HiMAP (Human Interactome Map) browser was used to explore the human literature-confirmed protein-protein interaction maps and the networks of interactions where the regulated genes are potentially involved. The analysis looked for the direct pathways connecting genes. The Biological Association Network of the downregulated genes included three major nodes; PECAM1 platelet/endothelial cell adhesion molecule 1 (PECAM1, also called CD31), selectin L (SELL, also called CD62L) and Fc fragment of low affinity IIIb, receptor (FCGR3B). Each of these genes interacts with other proteins (13, 12 and 7 interactions respectively; Figure 2B).

### Validation of microarray with real-time PCR

**Figure 3:**
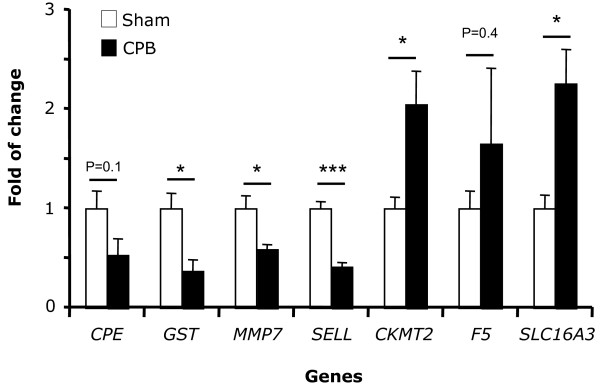
**Confirmation of the microarray results of changed genes in CPB versus Sham animals.** Changes in mRNA expression of *CPE*, *GST*, *MMP7*, *SELL*, *CKMT2*, *F5* and *SLC16A3* were verified in 10 pigs (5 CPB and 5 Sham; n = 5) by quantitative real-time-PCR. Results are shown as mean (±standard error of the mean, SEM) fold-change. *P < 0.05, ***P < 0.001.

Microarray results were confirmed by using real-time quantitative PCR on 7 selected genes that demonstrated the highest differential gene expression. The changes in expression levels of GST, MMP7, SELL, CKMT2 and SLC16A3 showed a very high degree of correlation with the microarray data (Figure 3). The other two examined genes showed a tendency to change similar to that of microarray experiment although these did not reach statistical significance (Figure 3).

### Validation of microarray with western blotting

**Figure 4:**
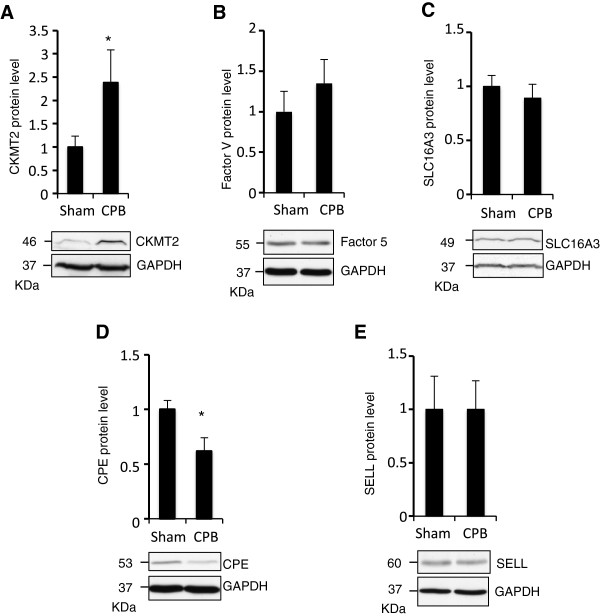
**Western blotting of identified gene products in kidney medulla of CPB and Sham animals.** Tissues from 10 pigs (5 CPB and 5 Sham; n = 5) were lysed to isolate protein content and Western blotting analysis performed probing for CKMT2 **(A)**, Factor 5 **(B)**, SLC16A3 **(C)**, CPE **(D)**, SELL **(E)**, and GAPDH. CKMT2 was significantly up-regulated in CPB compared to Sham samples. CPE was significantly down-regulated in CPB compared to Sham samples. CKMT2, Factor 5, SLC16A3, CPE and SELL bands were normalised to GAPDH levels. Data are mean ± SEM, * = p < 0.05.

Despite being greatly informative, changes in mRNA levels are insufficient to predict protein expression levels. We therefore went onto assessing the protein levels of some of the identified genes. We semi-quantitatively assessed protein levels in kidney medulla of CPB and Sham animals using Western blotting. CKMT2 and CPE showed significant increase and decrease in CPB compared to Sham samples respectively (Figure 4A, D). These protein expression changes were similar to those observed at the mRNA level. Factor 5, SLC16A3 and SELL showed no significant alteration in protein level following CPB (Figure 4B, C, E). However, we noted a tendency to increase by CPB for Factor 5.

## Discussion

This microarray-based gene expression profiling study confirms the existence of transcriptomic changes of the renal medulla in response to post-CPB AKI. The upregulated genes belonged to the metabolic process cluster. The downregulated genes belonged to cell adhesion/extracellular matrix and immune response clusters. Gene members of these clusters belonged to both injury and survival programmes. These changes indicate a mixed transcriptomic response of kidney’s medulla following CPB. It has been previously suggested that in response to early ischemia/reperfusion injury, a concomitant injuring and regenerating programmes of tubule cells are triggered [17]. This response of renal medullary tubular cells to ischemia implicates cell death, dedifferentiation of viable cells, proliferation, differentiation, and restitution of a normal epithelium [18, 19].

### Strengths and limitations of the study

The porcine model has several advantages over rodent models of acute kidney injury; there is significant homology between human and porcine renal anatomy, haemodynamics and function [20] and it is possible to examine the in vivo response to an injury that has direct clinical relevance such as CPB as well as any potential therapy. Previous studies have shown gene expression alterations in the kidney after ischemic AKI in murine and rat models [21–23]. However, findings in rodent models of AKI have failed to translate into clinical benefits principally due the poor homology between rodent and human renal anatomy and function, greater tolerance to ischaemia in humans and the difference in the nature of the experimental renal insult versus clinical scenarios associated with kidney injury [20]. Indeed, there are still questions regarding rodent models adequacy as a paradigm for human acute kidney injury [8]. This is the first study to examine gene expression change in the kidney of a large animal model that exhibits AKI in response to a common clinical injury; CPB. Indeed the porcine model used displays significant homology to cardiac surgery patients [13, 15]. This preliminary study is an important step towards improving our understanding of the molecular changes associated with post-CPB AKI, and helps developing reno-protective strategies in cardiac surgery.

Additionally, we elected to focus our analysis to the renal medulla because: 1. Pathological changes (ATN) in this anatomical region of the kidney have been part of the established paradigm of clinical AKI for many years [24]. 2. We failed to detect any ATN in this region of the kidney, as might have been expected on the basis of findings in rodent models, and in fact demonstrated that cellular ATP was preserved in the renal medulla despite the presence of refractory vasoconstriction, severe medullar hypoxia and diminished GRF [14]. 3. This region has fewer distinct anatomical compartments as compared to say the inner cortex where glomeruli and multiple proximal and distal tubular cell types are in close proximity, aiding interpretation.

A limitation of our study is that this model only reflects less severe AKI; the changes n renal clearance that we have described equate to the ‘Risk’ stage of the RIFLE classification, now considered as Stage 1 AKI in the recent Kidney Diseases Improving Global Outcomes consensus definition [25]. Animal recovery with re-evaluation at 24 hours is a key component, and strength of the current model as our previous studies have shown that changes in filtration immediately post CPB are not indicative of later pathophysiology [26, 27]. However reproducible recovery mandates that the nature of the injury sustained by study subjects is less severe. This limitation notwithstanding Stage 1 AKI has important prognostic implications in cardiac surgery. Moreover our model is unique in that it develops clinically defined AKI, as reflected by reductions in renal clearance, proteinuria and the release or the AKI biomarker IL-18 [28]. A second limitation is that alterations in gene expression identified by genechip analysis are not necessarily predictive of downstream functional and/or pathophysiologic networks. Thus, although we have confirmed an upregulation of select metabolic process genes and a down-regulation of extracellular matrix genes at the mRNA level and even at the protein level, multiple additional posttranscriptional and posttranslational events may be required to fully implicate these factors in the acute kidney injury following CPB.

### Translational relevance

Our data showed changes in gene expression of enzymes involved in metabolism. This finding is somewhat similar to previous studies [17, 29]. However, in our study, most metabolism cluster genes were upregulated, whereas metabolism genes were mostly downregulated by ischemia-reperfusion in the other studies [17, 29]. This could be explained by the difference in the model employed. Indeed, in our model, CPB was used to trigger kidney injury while direct renal ischemia-reperfusion was used in the other studies to initiate kidney injury [17, 29]. Additionally the mouse model expression studies used the whole kidney as opposed to our use of renal medulla in this study. Whereas rodent models typically demonstrate hypoxia, medullary ATP depletion, and ATN following warm ischaemia and reperfusion, this was not observed in our model. Indeed, in previous studies we have demonstrated increased medullary ATP levels, concomitant with hypoxia in the outer medulla and cortical vasoconstriction [14, 30]. This is perhaps attributable to the reduced solute delivery to the distal nephron attendant to acute reductions in GFR. An alternative explanation for these differences is that post CPB AKI is not characterised by ATN. This may also explain the failure of therapies developed in rodents to improve prognosis in cardiac surgery patients with AKI and highlights the value of large animal models as tools for translation.

In the validation experiments, gene expression alteration of selected genes by real-time PCR showed a very high degree of correlation with the microarray data suggesting that the changes of expression observed by genechip are most likely real. However the degree of correlation between the changes at the mRNA and protein levels was lower. This may be due to the common delay between transcription and protein synthesis.

The metabolism process enzyme with the largest observed upregulation was Acyl-CoA oxidase (ACOX3). ACOX3 is a key peroxisomal enzyme catalyzing the production of hydrogen peroxide during peroxisomal beta-oxidation of fatty acids. Fatty acid beta-oxidation and lipid peroxides are increased in chronic renal inflammation in rodents where farnesyl transferase inhibition modulates peroxisome enzyme activities thereby alleviating oxidative stress [31]. ACOX3 may therefore be important in redox signaling in the renal medulla after CPB, implying that oxidative stress may have a complex role in both injury and survival signaling in response to injury.

Another upregulated metabolism enzyme is Aconitase 1 (ACO1 or IRP1). ACO1 is a bifunctional protein that can exist as a functional cytosolic aconitase when it binds an iron-sulfur cluster, interconverting citrate to isocitrate, or as an apoprotein that can bind iron Responsive Elements to regulate the expression of mRNAs encoding proteins involved in iron homeostasis [32]. Changes in iron metabolism are central to cellular injury and survival in AKI [33]. A member of the monocarboxylate transporter family, solute carrier family-16 member 3 (SLC16A3), was also upregulated in the kidney medulla following CPB. SLC16 family members are involved in a wide range of pathways including energy metabolism, and are involved in the transport of lactic acid, pyruvate and ketones across the plasma membrane. Another member of the monocarboxylate transporter family, SLC16A7, was shown to be down regulated following renal ischemia-reperfusion in mouse [29], however as stated above this may be attributed to differences in the degree of injury and timing of the genomic analysis post injury.

Factor V (F5), a gene encoding an essential cofactor of the blood coagulation cascade, showed an upregulation following CPB. A recent study investigated the role of Factor V in diabetic nephropathy and demonstrated protective effect of factor V in diabetic nephropathy [34]. The increase of Factor V in our study could be part of a regenerating programme initiated in response to CPB.

CKMT2, a member of the creatine kinase isoenzyme family, showed a significant upregulation by CPB at the mRNA and protein levels. Mitochondrial creatine kinase (MtCK) is responsible for the transfer of high-energy phosphate from mitochondria to the cytosolic carrier, creatine. It has been shown that creatine kinase-deficient hearts exhibit increased susceptibility to ischemia-reperfusion injury and impaired calcium homeostasis [35], thus demonstrating a key role of an intact creatine kinase system for maintenance of Ca2+ homeostasis and withstanding ischemia-reperfusion injury. In our study, the increase of CKMT2 by CPB suggests a regenerating role for this factor.

The microarray data showed that Aldehyde dehydrogenase 4 (ALDH4A1) was increased 24 hours post CPB. It has been demonstrated that over-expression of ALDH4 in H1299 cells, resulted in significantly lower intracellular reactive oxygen species (ROS) levels than control cells after treatment with hydrogen peroxide or UV [36]. Additionally ALDH4 is a p53-induced gene with a protective role in cellular stress [36]. Therefore ALDH4A1 could play a regenerating role in our model.

Glutathione S-transferase alpha 2 (GST) gene expression showed the greatest relative reduction. Cytosolic and membrane-bound forms of GST function in the detoxification of carcinogens, therapeutic drugs, toxins and products of oxidative stress, by conjugation with glutathione. The alpha class of these enzymes also exhibit glutathione peroxidase activity thereby protecting the cells from reactive oxygen species and the products of peroxidation [37]. Reduced expression of GST in response to CPB may therefore indicate increased susceptibility to cellular injury. In an apparent paradox urine levels of GST have been identified as potential biomarkers of acute kidney injury. It is suggested that this reflects membrane permeability of damaged tubular cells, with some studies suggesting greater diagnostic accuracy for more severe AKI. In contrast the severity of AKI is mild to moderate in the current model and epithelial necrosis is not evident. We noted a decrease in Glutatione S-transferase (GSTA2) in response to CPB. However, Yoshida et al. reported an increase in GST following renal ischemia- reperfusion [29]. It is important to note that Yoshida et al. analyzed the whole kidney and not only the medulla as in our study.

The low affinity receptor for the Fc region of gamma immunoglobulins (IgG), FCGR3B, had a decreased expression following CPB-AKI. A recent study showed that impaired immune complex clearance arising from FCGR3B deficiency contributes to the pathology of systemic sclerosis, and FCGR3B copy number variation is a common risk factor for systemic autoimmunity [38]. Our data indicates that a reduction of FCGR3B expression may be involved in AKI injury following CPB.

Platelet/endothelial cell adhesion molecule 1 (PECAM1) was downregulated by CPB in this study. The encoded protein is a member of the immunoglobulin superfamily. In previous studies [14, 26] we have shown that loss of endothelial PECAM-1 expression, in association with reduced expression of other important endothelial cell-cell adhesion molecules is associated with endothelial and glycocalyceal injury. These changes are associated with endothelial permeability and dysfunction [14, 26].

Carboxypeptidase E (CPE) expression was decreased at the mRNA and protein level by CPB. Interestingly, CPE degradation contributes to palmitate-induced beta-cell ER stress and apoptosis [39]. Reduced CPE expression may therefore increase cellular susceptibility to injury.

PECAM1 interacts indirectly with CPE through THBS1-CD47 ligand receptor axis. The THBS1-CD47 axis controls a number of important cellular processes including production of reactive oxygen species. It has recently been suggested that CD47 promotes apoptotic cell death in renal IRI [40]. Additionally, CD47-dependent pathway is involved in platelet adhesion on inflamed vascular endothelium under flow [41]. Our data did not show expression changes of THBS1 and CD47 levels. However we noted a decrease of two factors, CPE and PECAM1 that interact with THBS1-CD47 axis.

The matrix metalloproteinase 7 (MMP-7) decreased by post-CPB AKI. MMP-7 is a protease that targets extracellular proteins [42] and is involved in tissue remodeling [43–46]. The reduction of MMP-7 expression could indicate a decrease in the remodeling capacity of the kidney following post CPB AKI.

Selectin L (SELL), a cell surface adhesion molecule, showed an expression decrease following CPB. Interestingly, it’s been demonstrated that transient expression of SELL in cardiac mesoangioblasts induced 2-fold increase in their transmigration and homing to the damaged heart [47] suggesting SELL implication in tissue repair. In our model, the expression decrease of SELL could indicate the kidney’s susceptibility to the injurious programme triggered by CPB.

## Conclusion

In conclusion, this study has characterised the transcriptomics changes in a model of post-CPB acute kidney injury with potential relevance to clinical settings. It has identified previously unreported complexity in the transcriptomic changes implicated in post-CPB kidney dysfunction. These differ significantly from rodent studies that typically evaluate genomic changes in entire kidneys, and in response to stimuli not commonly observed in clinical practice. Our observations highlight the value of large animal models in AKI research and provide insights into the failure of findings in rodent models to translate into clinical progress. Ultimately, our findings may contribute to the successful development of novel reno-protective strategies in cardiac surgery.

## Methods

Twelve adult, female, farm-bred, Large White-Landrace crossbred pigs weighing 50–70 kg were used in this pilot study. Animals received care in accordance with and under licence of the UK Home Office (Scientific Procedures) Act 1986. The investigation conforms to the Guide for the Care and Use of Laboratory Animals published by the US National Institutes of Health (NIH Publication No. 85–23, revised 1996). The study had received local (University of Bristol) institutional review board approval, and was conducted under UK Home Office License PPL 30/2522. The two groups received identical diet and unlimited access to water. Food, but not water, was restricted on the day of surgery.

### Intervention

We randomised animals (n = 6 per group) to: *Group 1.***Sham**, 2.5 hours under general anaesthesia with neck dissection, *Group 2.***CPB**, 2.5 hours of CPB via neck dissection, under general anaesthesia. A CPB time of 2.5 hours corresponds to prolonged CPB, a recognised risk factor for post cardiac surgery AKI that has been shown to result in significant kidney injury in our previous work [14, 30]. Sham animals underwent identical anaesthetic induction and maintenance and surgical dissection, and systemic heparinisation to those undergoing CPB. Anaesthesia and CPB were performed by a modification of our protocol described previously [14, 30]. Post procedure animals were recovered, re-anaesthetised and re-evaluated after 24 hours. Nephrectomy was performed prior to euthanasia.

### Acute kidney injury

#### Biochemical markers

^51^Cr-EDTA clearance was measured for 90 min pre-CPB and then at 90 min and 24 hours post CPB using single bolus ^51^Cr-EDTA injections with clearance determination obtained using the slope–intercept method as previously described [13]. Briefly, 4 ml of blood was extracted as a baseline measurement. Then 3.7 MBq of 51Cr-EDTA (Amersham, UK) was injected into a central vein. After injection, serial blood samples for analyses (4 ml) were then obtained over 90 min to calculate the plasma clearance curve. The blood was centrifuged (3000 × g for 10 min) and 3 ml of plasma extracted for scintillation counting of 51Cr-EDTA (gamma counter 15 min per sample). The radioactivity of the 51Cr-EDTA was measured together with a standard sample prepared in combination with that given to the pig. Clearance (Cl) of the marker was expressed as Cl = Q/AUC (ml min − 1), where Q = injected activity, AUC (total area under plasma clearance curve) = A/k1 + B/k2, where A and B are the zero time intercepts of the two exponentials and k1 and k2 the respective rate constants.

Creatinine clearance (CrCl), free water clearance and fractional sodium excretion were calculated from serum samples and urine samples taken over three time periods; 90 minutes pre-CPB; 90 minutes post weaning from CPB, and 90 minutes at 24 hours post CPB, using accepted formulae [48] as previously described [15].

Levels of IL-18, a specific marker of acute kidney injury detected in the urine >12 hours following renal injury [49], were measured in urine samples using ELISA (Bender MedSystems, Vienna, Austria). The urinary protein-to-creatinine ratio was determined by immunoturbidimetry on the Cobas Mira (Koni Inst, Sweden). Differences between groups were calculated using unpaired t-Tests using SPSS 14.0 (SPSS Inc, Chicago, Ill, USA). P values <0.05 were considered to be statistically significant.

#### Histological markers

Formalin fixed, paraffin embedded, 5 μm transverse renal sections stained with haematoxylin and eosin were scored for renal tubular injury and inflammation by an experienced renal pathologist blinded to the experimental conditions, as described previously [50, 51].

### Analysis of differentially expressed genes

To assess the effect of CPB on gene expression changes in the renal medulla, we compared pigs undergoing CPB versus a Sham group of pigs (n = 3 for each group). The porcine kidney was harvested via laparotomy, and immediately post ligation of the renal artery the organ was cut transversely into 8 roughly equal sections. Each section was then further sectioned into blocks of cortical, and outer medullary tissue each approximately 0.5 cm in diameter. The anatomical demarcations of these areas are very clear in fresh tissue, and the anatomical localisation of the biopsies has been confirmed in our previous studies. Each section was then placed in a cryotube, snap frozen in liquid nitrogen and stored at −80°C until analysis.

#### RNA extraction

Tissue was mechanically homogenized in lysis reagent (Qiagen, Crawley, UK) and Total RNA was purified with RNeasy Kit (Qiagen) and eluted into 30 μl of RNase-free water. The concentration and purity of the total RNA samples were assessed by spectrophotometry (Nanodrop, Wilmington, DE) and further analyzed for integrity with a Bioanalyzer 2100 with RNA 6000 Nano Assay (Agilent Technologies, Stockport, U.K.).

#### Gene microarrays

Kidney medulla total RNAs (1 μg) from individual animals were used to generate biotinylated cRNAs. The quantity and size distribution of purified cRNA was assessed on a Bioanalyzer 2100 using RNA 6000 Nano Assay (Agilent Technologies) to ensure that the cRNA amplification was successful. Target fragmentation was achieved by incubation at 94°C for 35 min in fragmentation buffer (40 mM Tris-acetate, pH 8.1/100 mM KOAc/30 mM MgOAc). The size distribution of the fragmented labelled transcripts was assessed on the Agilent Technologies Bioanalyzer 2100 using the RNA 6000 Nano Assay. These cRNAs samples were used for hybridisation to separate Affymetrix GeneChip arrays. For each experimental group, 3 samples from individual animals were processed. Hybridisation of the labelled cRNA to the Affymetrix GeneChip Porcine Genome Array was carried out for 16 h in the Affymetrix GeneChip Hybridization Oven 640. Then GeneChip arrays were stained and washed on the GeneChip Fluidics Station 450 (Affymetrix). The fluorescent signals were detected with an Affymetrix GeneChip Scanner 3000 and stored as high-resolution fluorescence intensity data file. These data were initially analysed with Affymetrix GeneChip operating software GCOS 1.2, which generates an expression report file that lists the quality control parameters. All of these parameters were scrutinized to ensure that array data had reached the necessary quality standards (scaling factor of <3-fold; average background values at 20–100 and the ratio of 3′:5′ signal no more than 3 for housekeeping genes GAPDH and β-actin). The complete MIAME-compliant datasets have been submitted and accepted by Gene Expression Omnibus at the National Center for Biotechnology Information; accession number GSE44782.

#### Microarray data analysis

Separate microarrays were probed with independently generated target from each tissue. Raw data (CEL files) were uploaded into ArrayStar software version 2.1 (DNASTAR) for normalization and statistical analysis. The robust multichip analysis (RMA) algorithm was used for background correction; quantile normalization and median polish summarization. The statistical analysis was carried out using ArrayStar software. A Student’s t-Test was used. *P* value was set to <0.05 and transcripts were filtered on the basis of ≥1.6-fold difference.

#### Gene annotations

All gene annotations were checked by using online tools and databases: Entrez Gene (http://www.ncbi.nlm.nih.gov). The DAVID (Database for Annotation, Visualization, and Integrated Discovery) resource [52–54] was used for functional annotation clustering. The HiMAP (Human Interactome Map) browser was used to explore the human literature-confirmed protein-protein interaction maps and the networks of interactions where the regulated genes are potentially involved.

#### cDNA synthesis and real-time PCR

Complementary DNA was reverse transcribed from 1 μg of total RNA using Superscript III cDNA first-strand synthesis kit (Invitrogen, UK), diluted 2-fold and 1 μl used in real-time PCR reactions. Primers for pig CPE, GST, MMP7, SELL, CKMT2, F5, and SLC16A3 were designed (Table 4) using the NCBI Primer-BLAST (Primer 3 and BLAST) resource and ordered from Sigma (UK). Amplification and detection of specific products were carried out with Roche Lightcycler 1.5 detection System. Each sample was performed in duplicate. 18S mRNA was used as endogenous control transcript in each sample. Relative expression ratios were calculated by the relative quantification real-time-PCR method [55]. Statistical analysis (unpaired t-test) was carried out using Instat 3 software (GraphPad Software, Inc, La Jolla, CA) and a *P* value was calculated for comparison. A *P* value of <0.05 was considered statistically significant.

**Table 4:** **Primers used in this study for real-time PCR**

Name	Sequence (5′- > 3′)
CPE-Forward	GCGCCGCGGTCAGCAGGATA
CPE-Reverse	AGGCTCACCCGGCTCGTGGA
GST-Forward	GGCAGCCAGAGGAAGCCTCCCA
GST-Reverse	AGAGGGTCCTGGGTGGCCCTG
MMP7-Forward	AGCAGCTATGCAGCTGGCCGT
MMP7-Reverse	GCCCTGAGCCTGTTCCCACTGC
SELL-Forward	GGGCGATGGGGAGCCCAACA
SELL-Reverse	GGCCACTGCATGACCTGGGCT
CKMT2-Forward	CACGCCGGCCATCTACGCCA
CKMT2-Reverse	GGGTGGCCGGGGTTGTCCAC
F5-Forward	TGGGGTGGTGACGGCAGGGA
F5-Reverse	GCCCAGCTGGTGCCCAGGAC
SLC16A3-Forward	AGGACGGGGAGCTCGTGGCA
SLC16A3-Reverse	CCACCGCGGGGCTTGAGGAC

#### Western blotting

Protein levels in renal tissue were quantified by Western blotting as previously described [56]. Briefly, total protein extracts were prepared from kidney medulla samples, separated on SDS polyacrylamide gel (30 μg of protein extracts) and transferred to HybondTM nitrocellulose membrane (Amersham). Blocked membranes were incubated in primary polyclonal antibodies (rabbit anti-CPE, rabbit anti- SELL, rabbit anti-CKMT2, rabbit anti-SLC16A3, Abcam) and monoclonal mouse anti-Factor 5 (Abcam), washed and incubated in HRP-coupled anti-rabbit or anti-mouse secondary antibodies (Amersham). Membranes were exposed to HyperfilmTM (Amersham) and protein bands were quantified using NIH Image J software. Statistical analysis (unpaired t-test) was carried out using Instat 3 software (GraphPad Software, Inc, La Jolla, CA) and a *P* value was calculated for comparison. A *P* value of <0.05 was considered statistically significant.

## Electronic supplementary material

Additional file 1: Figure S1: Graphs showing effect of 2.5 hours of cardiopulmonary bypass or sham intervention on A. Cr51 EDTA Clearance and B. Calculated Creatinine Clearance, pre intervention (Pre), and at 1.5 hours and 24 hours post intervention. Values represent mean (S.E.M). *p < 0.01. For graphs pooled estimates for pairwise comparisons derived from Analysis of Variance for Repeated Measures with adjustment for baseline Cr51 EDTA Clearance estimated at 138 ml/min and Creatinine Clearance estimated at 113 ml/min, were as follows: Cr51 EDTA; Sham-CPB 23.5 ml/min (95%CI 3.1 to 4.0 ml/min), test for overall treatment effect p = 0.028, test for time*group interaction p = 0.789. Creatinine Clearance; Sham--‒CPB 54.1 ml/min (95%CI 22.2 to 86.0), test for overall treatment effect of group p = 0.004, test for group*time interaction p = 0.073. C. demonstrates close correlation between measured Cr51 EDTA clearance and calculated creatinine clearance. (PDF 116 KB)
