# Supplementary material for: Changes in renal medulla gene expression in a pre-clinical model of post cardiopulmonary bypass acute kidney injury
Source: BMC Genomics. 2014 Oct 21;15(1):916. doi: 10.1186/1471-2164-15-916 (PMC4210505; doi:10.1186/1471-2164-15-916)
Supplement: Supplementary file 1 — Additional file 1: Figure S1: Graphs showing effect of 2.5 hours of cardiopulmonary bypass or sham intervention on A. Cr51 EDTA Clearance and B. Calculated Creatinine Clearance, pre intervention (Pre), and at 1.5 hours and 24 hours post intervention. Values represent mean (S.E.M). *p < 0.01. For graphs pooled estimates for pairwise comparisons derived from Analysis of Variance for Repeated Measures with adjustment for baseline Cr51 EDTA Clearance estimated at 138 ml/min and Creatinine Clearance estimated at 113 ml/min, were as follows: Cr51 EDTA; Sham-CPB 23.5 ml/min (95%CI 3.1 to 4.0 ml/min), test for overall treatment effect p = 0.028, test for time*group interaction p = 0.789. Creatinine Clearance; Sham--‒CPB 54.1 ml/min (95%CI 22.2 to 86.0), test for overall treatment effect of group p = 0.004, test for group*time interaction p = 0.073. C. demonstrates close correlation between measured Cr51 EDTA clearance and calculated creatinine clearance. (PDF 116 KB) [file 12864_2014_6603_MOESM1_ESM.pdf]

eFigure 1. Graphs showing effect of 2.5 hours of cardiopulmonary bypass or sham intervention on **A.** Cr51 EDTA Clearance and **B.** Calculated Creatinine Clearance, pre intervention (Pre), and at 1.5 hours and 24 hours post intervention. Values represent mean (S.E.M). * p<0.01.

For graphs pooled estimates for pairwise comparisons derived from Analysis of Variance for Repeated Measures with adjustment for baseline Cr51 EDTA Clearance estimated at 138ml/min and Creatinine Clearance estimated at 113ml/min, were as follows: Cr51 EDTA; Sham-CPB 23.5ml/min (95%CI 3.1 to 44.0ml/min), test for overall treatment effect p=0.028, test for time*group interaction p=0.789. Creatinine Clearance; Sham-CPB 54.1 ml/min (95%CI 22.2 to 86.0), test for overall treatment effect of group p=0.004, test for group* time interaction p=0.073. **eFigure 1C.** demonstrates close correlation between measured Cr51 EDTA clearance and calculated creatinine clearance.

A

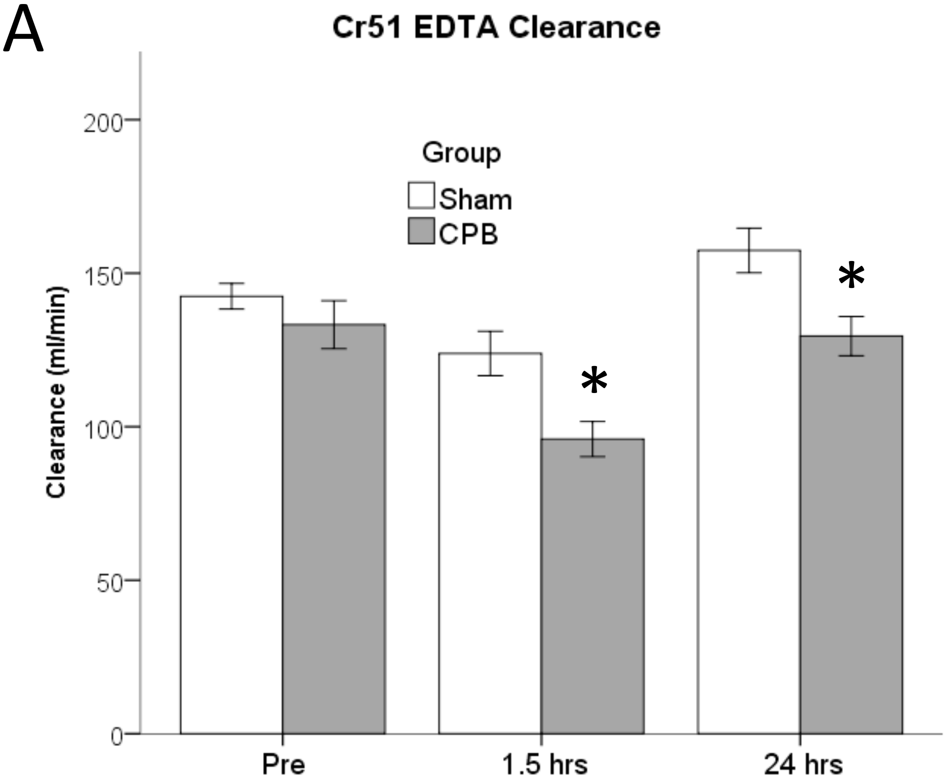

B

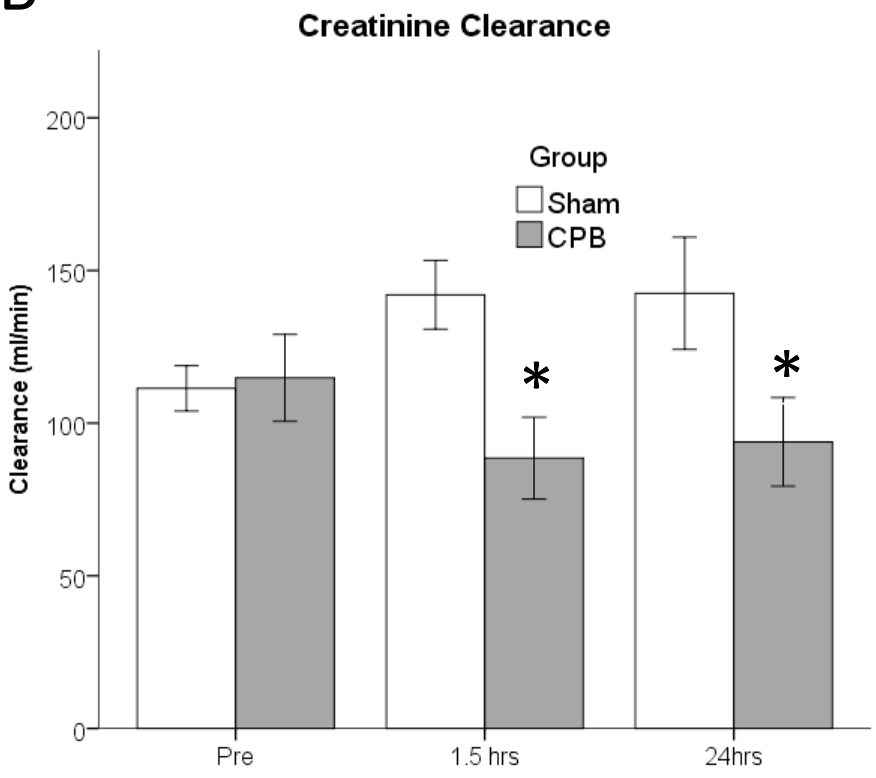

Creatinine Clearance
Clearance (ml/min)
200
150
100
50
0
Group
Sham
CPB
*
*
Pre
1.5 hrs
24hrs

C

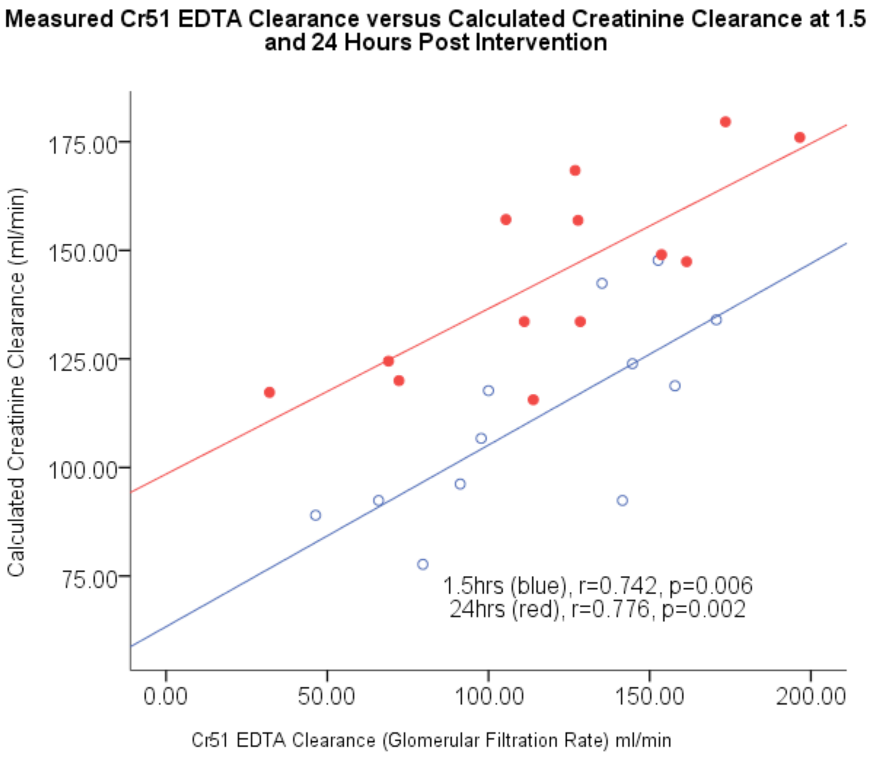

Measured Cr51 EDTA Clearance versus Calculated Creatinine Clearance at 1.5 and 24 Hours Post Intervention
Calculated Creatinine Clearance (ml/min)
175.00
150.00
125.00
100.00
75.00
1.5hrs (blue), r=0.742, p=0.006
24hrs (red), r=0.776, p=0.002
0.00
50.00
100.00
150.00
200.00
Cr51 EDTA Clearance (Glomerular Filtration Rate) ml/min
